# RETRACTION: Human Umbilical Cord Mesenchymal Stem Cell‐Derived Exosomes Combined With Gelatin Methacryloyl Hydrogel to Promote Fractional Laser Injury Wound Healing

**DOI:** 10.1111/iwj.70567

**Published:** 2025-04-18

## Abstract

**RETRACTION:** ZhangX. , DingP. , ChenY. , LinZ. , ZhaoX. , and XieH. , “Human Umbilical Cord Mesenchymal Stem Cell‐Derived Exosomes Combined With Gelatin Methacryloyl Hydrogel to Promote Fractional Laser Injury Wound Healing,” International Wound Journal 20, no. 10 (2023): 4040–4049, 10.1111/iwj.14295.37429607 PMC10681517

The above article, published online on 10 July 2023, in Wiley Online Library (http://onlinelibrary.wiley.com/), has been retracted by agreement between the journal Editor in Chief, Professor Keith Harding; and John Wiley & Sons Ltd. Following an investigation by the publisher, all parties have concluded that this article was accepted solely on the basis of a compromised peer review process. The editors have therefore decided to retract the article. The authors did not respond to our notice regarding the retraction.

**RETRACTION:** X. Zhang , P. Ding , Y. Chen , Z. Lin , X. Zhao , and H. Xie , “Human Umbilical Cord Mesenchymal Stem Cell‐Derived Exosomes Combined With Gelatin Methacryloyl Hydrogel to Promote Fractional Laser Injury Wound Healing,” International Wound Journal 20, no. 10 (2023): 4040–4049, 10.1111/iwj.14295.37429607 PMC10681517

The above article, published online on 10 July 2023, in Wiley Online Library (http://onlinelibrary.wiley.com/), has been retracted by agreement between the journal Editor in Chief, Professor Keith Harding; and John Wiley & Sons Ltd. Following an investigation by the publisher, all parties have concluded that this article was accepted solely on the basis of a compromised peer review process. The editors have therefore decided to retract the article. The authors did not respond to our notice regarding the retraction.

